# Supplementary material for: Over half of clinical practice guidelines use non-systematic methods to inform recommendations: A methods study
Source: PLoS One. 2021 Apr 22;16(4):e0250356. doi: 10.1371/journal.pone.0250356 (PMC8062080; doi:10.1371/journal.pone.0250356)
Supplement: S1 Appendix — (DOCX) [file pone.0250356.s001.docx]

**S1 Appendix. Search Strategies.**

Date searched: January 8, 2019

*Epistemonikos*

Dates searched: January 1, 2017 to December 31, 2018

Limit: Broad syntheses

*Turning Research Into Practice (TRIP)*

Dates searched: January 1, 2017 to December 31, 2018

Limit: None
