# Supplementary figures and images for: Over half of clinical practice guidelines use non-systematic methods to inform recommendations: A methods study
Source: PLoS One. 2021 Apr 22;16(4):e0250356. doi: 10.1371/journal.pone.0250356 (PMC8062080; doi:10.1371/journal.pone.0250356)

**S2 Appendix. Flowchart of the study selection process.**


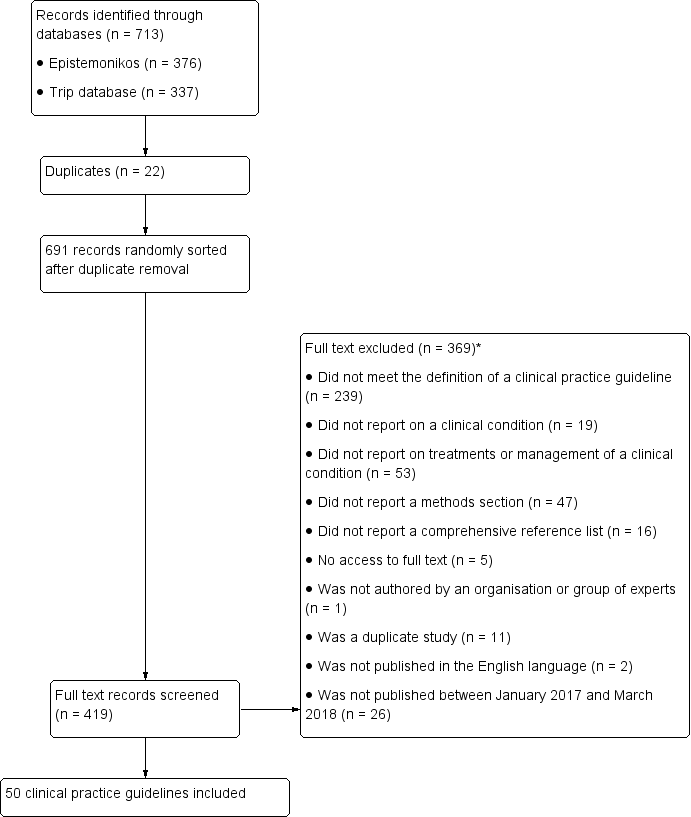

Supplement: S2 Appendix — (DOCX) [file pone.0250356.s002.docx]
