# Supplementary material for: Over half of clinical practice guidelines use non-systematic methods to inform recommendations: A methods study
Source: PLoS One. 2021 Apr 22;16(4):e0250356. doi: 10.1371/journal.pone.0250356 (PMC8062080; doi:10.1371/journal.pone.0250356)
Supplement: S3 Appendix — (DOCX) [file pone.0250356.s003.docx]

**S3 Appendix. Included Clinical practice guidelines.**

1. Addington J, Addington D, Abidi S, Raedler T, Remington G. Canadian Treatment Guidelines for Individuals at Clinical High Risk of Psychosis. Can J Psychiatry. 2017;62(9):656-61.

2. Allan GM, Ramji J, Perry D, Ton J, Beahm NP, Crisp N, et al. Simplified guideline for prescribing medical cannabinoids in primary care. Can Fam Physician. 2018;64(2):111-20.

3. Badhwar V, Rankin JS, Damiano RJ, Jr., Gillinov AM, Bakaeen FG, Edgerton JR, et al. The Society of Thoracic Surgeons 2017 Clinical Practice Guidelines for the Surgical Treatment of Atrial Fibrillation. Ann Thorac Surg. 2017;103(1):329-41.

4. Baumgartner H, Falk V, Bax JJ, De Bonis M, Hamm C, Holm PJ, et al. 2017 ESC/EACTS Guidelines for the management of valvular heart disease. Eur Heart J. 2017;38(36):2739-91.

5. Braga LH, Lorenzo AJ, Romao RLP. Canadian Urological Association-Pediatric Urologists of Canada (CUA-PUC) guideline for the diagnosis, management, and followup of cryptorchidism. Can Urol Assoc J. 2017;11(7):E251-e60.

6. British Columbia Centre for Substance Abuse (BCCSU). A Guideline for the Clinical Management of Opioid Use Disorder. https://www.bccsu.ca/wp-content/uploads/2017/06/BC-OUD-Guidelines_June2017.pdf. BC, Canada: BC Ministry of Health; 2017.

7. British Columbia Centre for Substance Abuse (BCCSU). A guideline for the clinical management of opioid use disorder. In: Committee POUDTG, editor. Vancouver, Canada. https://www.bccsu.ca/wp-content/uploads/2017/06/BC-OUD-Guidelines_June2017.pdf: British Columbia Centre on Substance Use; 2017.

8. Brooks AJ, Smith PJ, Cohen R, Collins P, Douds A, Forbes V, et al. UK guideline on transition of adolescent and young persons with chronic digestive diseases from paediatric to adult care. Gut. 2017;66(6):988-1000.

9. Chang SS, Bochner BH, Chou R, Dreicer R, Kamat AM, Lerner SP, et al. Treatment of Non-Metastatic Muscle-Invasive Bladder Cancer: AUA/ASCO/ASTRO/SUO Guideline. J Urol. 2017;198(3):552-9.

10. Chin J, Rumble RB, Kollmeier M, Heath E, Efstathiou J, Dorff T, et al. Brachytherapy for Patients With Prostate Cancer: American Society of Clinical Oncology/Cancer Care Ontario Joint Guideline Update. Journal of clinical oncology : official journal of the American Society of Clinical Oncology. 2017;35(15):1737-43.

11. Corcos J, Przydacz M, Campeau L, Gray G, Hickling D, Honeine C, et al. CUA guideline on adult overactive bladder. Can Urol Assoc J. 2017;11(5):E142-e73.

12. Dreyling M, Campo E, Hermine O, Jerkeman M, Le Gouill S, Rule S, et al. Newly diagnosed and relapsed mantle cell lymphoma: ESMO Clinical Practice Guidelines for diagnosis, treatment and follow-up. Ann Oncol. 2017;28(suppl_4):iv62-iv71.

13. Duceppe E, Parlow J, MacDonald P, Lyons K, McMullen M, Srinathan S, et al. Canadian Cardiovascular Society Guidelines on Perioperative Cardiac Risk Assessment and Management for Patients Who Undergo Noncardiac Surgery. Can J Cardiol. 2017;33(1):17-32.

14. Duggal M, Tong HJ, Al-Ansary M, Twati W, Day PF, Nazzal H. Interventions for the endodontic management of non-vital traumatised immature permanent anterior teeth in children and adolescents: a systematic review of the evidence and guidelines of the European Academy of Paediatric Dentistry. Eur Arch Paediatr Dent. 2017;18(3):139-51.

15. European Society for Medical Oncology (ESMO) Guidelines Committee. eUpdate–Renal Cell Carcinoma Treatment Recommendations. European Society for Medical Oncology; 2017a.

16. European Society for Medical Oncology (ESMO) Guidelines Committee. eUpdate– Chronic Lymphocytic Leukaemia Treatment Recommendations. Lugano, Switzerland: ESMO. https://www.esmo.org/Guidelines/Haematological-Malignancies/Chronic-Lymphocytic-Leukaemia/eUpdate-Treatment-Recommendations; 2017b.

17. Finelli A, Ismaila N, Bro B, Durack J, Eggener S, Evans A, et al. Management of small renal masses: American Society of Clinical Oncology clinical practice guideline. 2017;35(6):668-80.

18. Franz MJ, MacLeod J, Evert A, Brown C, Gradwell E, Handu D, et al. Academy of Nutrition and Dietetics Nutrition Practice Guideline for Type 1 and Type 2 Diabetes in Adults: Systematic Review of Evidence for Medical Nutrition Therapy Effectiveness and Recommendations for Integration into the Nutrition Care Process. J Acad Nutr Diet. 2017;117(10):1659-79.

19. Frost JL, Campos-Outcalt D, Hoelting D. Pharmacologic management of newly detected atrial fibrillation. April 2017. https://www.aafp.org/dam/AAFP/documents/patient_care/clinical_recommendations/a-fib-guideline.pdf: American Academy of Family Physicians; 2017.

20. Fung KFK, Eason E. No. 133-Prevention of Rh Alloimmunization. Journal of obstetrics and gynaecology Canada : JOGC = Journal d'obstetrique et gynecologie du Canada : JOGC. 2018;40(1):e1-e10.

21. Gordon C, Amissah-Arthur MB, Gayed M, Brown S, Bruce IN, D'Cruz D, et al. The British Society for Rheumatology guideline for the management of systemic lupus erythematosus in adults: Executive Summary. Rheumatology (Oxford). 2018;57(1):14-8.

22. Hadji P, Aapro MS, Body JJ, Gnant M, Brandi ML, Reginster JY, et al. Management of Aromatase Inhibitor-Associated Bone Loss (AIBL) in postmenopausal women with hormone sensitive breast cancer: Joint position statement of the IOF, CABS, ECTS, IEG, ESCEO IMS, and SIOG. J Bone Oncol. 2017;7:1-12.

23. Hadjipanayis CG, Carlson ML, Link MJ, Rayan TA, Parish J, Atkins T, et al. Congress of Neurological Surgeons Systematic Review and Evidence-Based Guidelines on Surgical Resection for the Treatment of Patients With Vestibular Schwannomas. Neurosurgery. 2018;82(2):E40-e3.

24. Hanley J, McKernan A, Creagh MD, Classey S, McLaughlin P, Goddard N, et al. Guidelines for the management of acute joint bleeds and chronic synovitis in haemophilia: A United Kingdom Haemophilia Centre Doctors' Organisation (UKHCDO) guideline. Haemophilia. 2017;23(4):511-20.

25. Ho VP, Patel NJ, Bokhari F, Madbak FG, Hambley JE, Yon JR, et al. Management of adult pancreatic injuries: A practice management guideline from the Eastern Association for the Surgery of Trauma. J Trauma Acute Care Surg. 2017;82(1):185-99.

26. Isaacs CG, Kistler, C., Hunold, K.M., Pereira, G.F., Buchbinder, M., Weaver, M.A., Doherty, S., Knott, J., Bennetts, S., Jazayeri, M., Huckson, S. VA/DoD clinical practice guideline for opioid therapy for chronic pain. Department of Veterans Affairs. Department of Defense. https://pami.emergency.med.jax.ufl.edu/wordpress/files/2019/07/Basic-of-Pain-References-07012019.pdf; 2017.

27. KDIGO Update Work Group. KDIGO 2017 Clinical Practice Guideline Update for the Diagnosis, Evaluation, Prevention, and Treatment of Chronic Kidney Disease-Mineral and Bone Disorder (CKD-MBD). Kidney Int Suppl. 2017;7(1):1-59.

28. Kusumoto FM, Bailey KR, Chaouki AS, Deshmukh AJ, Gautam S, Kim RJ, et al. Systematic review for the 2017 AHA/ACC/HRS guideline for management of patients with ventricular arrhythmias and the prevention of sudden cardiac death: A Report of the American College of Cardiology/American Heart Association Task Force on Clinical Practice Guidelines and the Heart Rhythm Society. Heart Rhythm. 2018;15(10):e253-e74.

29. Lutz S, Balboni, T., Jones, J., Lo, S., Petit, J., Rich, S. E., Wong, R., Hahn, C. Palliative radiation therapy for bone metastases: Update of an ASTRO Evidence-Based Guideline. Pract Radiat Oncol. 2017;7(1):4-12.

30. Millington GWM, Collins A, Lovell CR, Leslie TA, Yong ASW, Morgan JD, et al. British Association of Dermatologists' guidelines for the investigation and management of generalized pruritus in adults without an underlying dermatosis, 2018. Br J Dermatol. 2018;178(1):34-60.

31. Moreau P, San Miguel J, Sonneveld P, Mateos MV, Zamagni E, Avet-Loiseau H, et al. Multiple myeloma: ESMO Clinical Practice Guidelines for diagnosis, treatment and follow-up. Ann Oncol. 2017;28 Suppl 4:iv52-iv61.

32. Mowery NT, Bruns BR, MacNew HG, Agarwal S, Enniss TM, Khan M, et al. Surgical management of pancreatic necrosis: A practice management guideline from the Eastern Association for the Surgery of Trauma. J Trauma Acute Care Surg. 2017;83(2):316-27.

33. Mulhall JP, Trost LW, Brannigan RE, Kurtz EG, Redmon JB, Chiles KA, et al. Evaluation and Management of Testosterone Deficiency: AUA Guideline. J Urol. 2018;200(2):423-32.

34. National GAU, . Cystic Fibrosis: Diagnosis and management. https://www.ncbi.nlm.nih.gov/books/NBK464183/pdf/Bookshelf_NBK464183.pdf: National Institute for Health and Care Excellence; 2017a.

35. National Guideline Alliance (UK). Eating Disorders: Recognition and Treatment. NICE Guideline, No. 69. London, UK: National Institute for Health and Care Excellence; 2017b.

36. Panel on Antiretroviral Therapy and Medical Management of Children Living with HIV. Guidelines for the use of antiretroviral agents in pediatric HIV infection. 2017. US Office of AIDS Research Advisory Council (OARAC). https://aidsinfo.nih.gov/guidelines/html/2/pediatric-arv/45/whats-new-in-the-guidelines

https://aidsinfo.nih.gov/contentfiles/lvguidelines/PediatricGuidelines.pdf 2017.

37. Practice Committee of the American Society for Reproductive Medicine. Role of metformin for ovulation induction in infertile patients with polycystic ovary syndrome (PCOS): a guideline. Fertil Steril. 2017;108(3):426-41.

38. Qaseem A, Barry MJ, Humphrey LL, Forciea MA. Oral Pharmacologic Treatment of Type 2 Diabetes Mellitus: A Clinical Practice Guideline Update From the American College of Physicians. Ann Intern Med. 2017;166(4):279-90.

39. Registered Nurses Association of Ontario (RNAO). Integrating tobacco interventions into daily practice. Toronto (ON): Registered Nurses Association of Ontario (RNAO). https://www.guidelinecentral.com/summaries/integrating-tobacco-interventions-into-daily-practice/#section-442; 2017.

40. Roberts G, Pfaar O, Akdis CA, Ansotegui IJ, Durham SR, Gerth van Wijk R, et al. EAACI Guidelines on Allergen Immunotherapy: Allergic rhinoconjunctivitis. Allergy. 2018;73(4):765-98.

41. Roselló S, Blasco I, García Fabregat L, Cervantes A, Jordan K. Management of infusion reactions to systemic anticancer therapy: ESMO Clinical Practice Guidelines. Ann Oncol. 2018;29(Suppl 4):iv260.

42. Rosen R, Vandenplas Y, Singendonk M, Cabana M, DiLorenzo C, Gottrand F, et al. Pediatric Gastroesophageal Reflux Clinical Practice Guidelines: Joint Recommendations of the North American Society for Pediatric Gastroenterology, Hepatology, and Nutrition and the European Society for Pediatric Gastroenterology, Hepatology, and Nutrition. Journal of pediatric gastroenterology and nutrition. 2018;66(3):516-54.

43. Sateia MJ, Buysse DJ, Krystal AD, Neubauer DN, Heald JL. Clinical Practice Guideline for the Pharmacologic Treatment of Chronic Insomnia in Adults: An American Academy of Sleep Medicine Clinical Practice Guideline. J Clin Sleep Med. 2017;13(2):307-49.

44. Saxby N, Painter C, Kench A, King S, Crowder T, van der Haak N. Nutrition guidelines for cystic fibrosis in Australia and New Zealand. Sydney: thoracic society of Australia and New Zealand. 2017.

45. Schmidt-Hieber M, Bierwirth J, Buchheidt D, Cornely OA, Hentrich M, Maschmeyer G, et al. Diagnosis and management of gastrointestinal complications in adult cancer patients: 2017 updated evidence-based guidelines of the Infectious Diseases Working Party (AGIHO) of the German Society of Hematology and Medical Oncology (DGHO). Annals of hematology. 2018;97(1):31-49.

46. Tan DHS, Hull MW, Yoong D, Tremblay C, O'Byrne P, Thomas R, et al. Canadian guideline on HIV pre-exposure prophylaxis and nonoccupational postexposure prophylaxis. Cmaj. 2017;189(47):E1448-e58.

47. Van Poznak C, Somerfield MR, Barlow WE, Biermann JS, Bosserman LD, Clemons MJ, et al. Role of Bone-Modifying Agents in Metastatic Breast Cancer: An American Society of Clinical Oncology-Cancer Care Ontario Focused Guideline Update. Journal of clinical oncology : official journal of the American Society of Clinical Oncology. 2017;35(35):3978-86.

48. Virgo KS, Basch E, Loblaw DA, Oliver TK, Rumble RB, Carducci MA, et al. Second-Line Hormonal Therapy for Men With Chemotherapy-Naive, Castration-Resistant Prostate Cancer: American Society of Clinical Oncology Provisional Clinical Opinion. Journal of clinical oncology : official journal of the American Society of Clinical Oncology. 2017;35(17):1952-64.

49. Wessells H, Angermeier KW, Elliott S, Gonzalez CM, Kodama R, Peterson AC, et al. Male Urethral Stricture: American Urological Association Guideline. J Urol. 2017;197(1):182-90.

50. World Health Organization. WHO guidelines on integrated care for older people (ICOPE). WHO World Health Organization, Geneva. 2017.
