# Supplementary material for: Over half of clinical practice guidelines use non-systematic methods to inform recommendations: A methods study
Source: PLoS One. 2021 Apr 22;16(4):e0250356. doi: 10.1371/journal.pone.0250356 (PMC8062080; doi:10.1371/journal.pone.0250356)
Supplement: S4 Appendix — (DOCX) [file pone.0250356.s004.docx]

**S4 Appendix. Table of associations.**

**Table of chi-square test of independence** **results**

| **Predictor variable** | **Dependent variable** | **Proportion yes** | **Chi squared results** | **P value** |
| --- | --- | --- | --- | --- |
| GRADE compliant | Funder (all) |  | 9.491 | 0.050 |
|  | - Funder: government | 12% | 4.48 | 0.034 |
|  | - Funder: Medical society or association | 12% | 0.09 | 0.768 |
|  | - Funder: pharmaceutical industry | 0% | 6.17 | 0.013 |
|  | - No funding | 4% | 0.02 | 0.880 |
|  | - Funding source not reported | 8% | 0.81 | 0.368 |
|  | Scope (narrow, broad) | 22% | 1.4 | 0.20 |
|  | Conflict of Interest | 34% | 0.18 | 0.07 |
|  | Affiliation with the pharmaceutical industry | 18% | 2.4 | 0.10 |
|  | Continent (all)3 |  | 6.0 | 0.05 |
|  | - International | 6% | 5.674 | 0.017 |
|  | - Europe | 8% | 1.2357 | 0.266 |
|  | - North America | 22% | 0.014468 | 0.904 |
| Systematic process used | Funder (all)2 |  | 3.60 | 0.463 |
|  | - Government | 8% | 0.7811 | 0.377 |
|  | - Medical society or association | 10% | 0.23042 | 0.631 |
|  | - Pharmaceutical industry | 2% | 2.2009 | 0.138 |
|  | - No funding | 6% | 1.0152 | 0.314 |
|  | - Not reported | 6% | 0.1324 | 0.716 |
|  | Scope (narrow, broad) | 16% | 0 | 1.0 |
|  | Affiliation with the pharmaceutical industry | 18% | 0.6134 | 0.433 |
|  | Conflict of Interest | 32% | 0.9804 | 0.322 |
|  | Continent (all)3 |  | 7.551 | 0.023 |
|  | - International | 6% | 6.782 | 0.009 |
|  | - Europe | 10% | 0.006082 | 0.938 |
|  | - North America | 14% | 2.5888 | 0.108 |

2: Df=4, 3: Df=2
