# Supplementary material for: Over half of clinical practice guidelines use non-systematic methods to inform recommendations: A methods study
Source: PLoS One. 2021 Apr 22;16(4):e0250356. doi: 10.1371/journal.pone.0250356 (PMC8062080; doi:10.1371/journal.pone.0250356)
Supplement: Attachment — Submitted filename: Response to Reviewers 20210329.docx [file pone.0250356.s005.docx]

Plos ONE: 'Response to Reviewers'

Editor comments:

1. In the light that the search strategy is more than two years old, we think that it is important to discuss the current relevance and how the field may have evolved since 2018.

- We randomly sampled CPGs from TRIP and Epistemonikos databases from 2017 and 2018. We believe, even though our search range is now 3 years old that the reporting and methods used in CPGs would not have changed substantially. Notably no changes have been made to the AGREE II tool which is used to assess CPG reporting, quality and processes (Kredo 2016).
- “However, as we only included guidelines published between 2017 and 2018, in English, and indexed in TRIP and Epistemonikos, our findings can neither be generalized to CPGs published in another time period, language or to guidelines indexed elsewhere.”
- We have included this statement of acknowledgement of this limitation in our limitations section.

Kredo T, Bernhardsson S, Machingaidze S, Young T, Louw Q, Ochodo E, Grimmer K. Guide to clinical practice guidelines: the current state of play. Int J Qual Health Care. 2016 Feb;28(1):122-8. doi: 10.1093/intqhc/mzv115. Epub 2016 Jan 20. PMID: 26796486; PMCID: PMC4767049.

2. In addition, it might be worth to discuss the future impact of the Right-Statement.

- We have added the following to the discussion:

“Efforts to improve reporting are underway with the publication of the RIGHT statement for reporting clinical practice guidelines {Chen, 2017 #504}. Two of the 34 items in the RIGHT checklist ask about systematic review methods, namely:

- 11a. Indicate whether the guideline is based on new systematic reviews done specifically for this guideline or whether existing systematic reviews were used.
- 11b. If the guideline developers used existing systematic reviews, reference these and describe how those reviews were identified and assessed (provide the search strategies and the selection criteria, and describe how the risk of bias was evaluated) and whether they were updated.

The RIGHT statement, while providing a minimum standard of what should be reported in a clinical practice guideline, also goes further by suggesting methods that should have been conducted (i.e. doing a systematic review, or used existing systematic review). These reporting recommendation will help knowledge users identify what processes were followed by guideline developers to gather, assess and synthesise evidence.”

3. If you would like to make changes to your financial disclosure, please include your updated statement in your cover letter.

- We have no funding for this study, and have no financial conflicts of interest.

4. Please ensure that your manuscript meets PLOS ONE's style requirements, including those for file naming.

- We have made edits to the manuscript based on a review of the style requirements: namely, double spacing, line numbering, added acknowledgements, removed figures and put into separate files.

5. Please review your reference list to ensure that it is complete and correct.

- We reviewed the references and found a few errors. Thanks for brining this to our attention.

6. We noticed you have some minor occurrence of overlapping text with the following previous publication, which needs to be addressed:

<https://pubmed.ncbi.nlm.nih.gov/31964662/>

The text that needs to be addressed involves the Strengths and Limitations section and the caption of Figure 1.

In your revision ensure you cite all your sources (including your own works), and quote or rephrase any duplicated text outside the methods section. Given the extent of the overlap in the methods section, we ask that you make sure any directly reproduced text is quoted and cited clearly.

- I have reworded the Strengths and Limitations, and caption and title of Figure 1, and added a reference to our protocol (Lunny 2020).
- We have rewritten the methods section to avoid duplicated text.

**Reviewer #1: Peer review comments**
Thank you for the opportunity to review this interesting manuscript on guideline development. The authors have found that there are less guidelines using systematic methods in their development than their should be and other critical issues in guideline development. I thank the authors for conducting this work and have made some suggestions below.

- Thank you!

Minor
I think the statement regarding optimal methods for guideline development are lacking is somewhat misleading. Although different development organisations may have slight differences, there is consensus surrounding key aspects of guideline development (particularly in relation to the use of systematic reviews to inform their development) as evidenced by the IOM definition, the GIN standards, the GRADE approach, the GIN McMaster checklist, NHMRC guidelines for guidelines, NICE, AGREE II etc, etc. there are also reporting standards (RIGHT) for guidelines. I think the findings from this study are more surprising that DESPITE these standards and guidance, there are still many who do not use evidence synthesis.

- Thank you for your comment, and we agree with you. We have amended our sentence “Although standards for CPG development are published [4-6], consensus about the optimal methods for their development is lacking [3].” To “Standards for guideline development are published by many organisations [4-6], and although there are slight differences, consensus exists surrounding key aspects of guideline development particularly regarding the use of systematic reviews to inform their development. Despite these standards and guidance, many guidelines do not conduct a systematic, evidence-based approach to evidence synthesis [1, 2].”

A bit more information on selecting the sample size would be useful.

- We chose this sample size so that it would be large enough to include a variety of clinical conditions and be feasible for two reviewers to extract and assess reporting in the time available to the research team.
- We also added this sentence to the search (section 2.2), “This time period was selected to limit the number of guidelines screened due to resource limitations,” and section 2.3 “We chose this sample size as it was large enough to include a variety of clinical conditions, and be feasible for two reviewers to extract and assess reporting in the time available to the research team.”

**Reviewer #2**

The publication by Lunny et al deals with a relevant topic. Although quality criteria and methodological standards for guidelines have been established for several decades, guidelines still exist that do not fully comply with these requirements and whose recommendations are not always consistently based on the necessary evidence or do not always adequately evaluate and interpret it.

The present study is based on transparent and comprehensible methods in terms of research, inclusion criteria, data extraction and analysis. It should be critically noted that due to the selection of sources for the search, the proportion of guidelines from the USA and Canada is very high in relation to guidelines from other countries. Concomitantly, non-English language guidelines are not analyzed, although many countries (including Germany) have well-developed guideline programs that result in evidence-based guidelines.

- Thank you for your feedback. We included guidelines in any language, however, because we searched only TRIP and Epistemonikos, we retrieved only English language guidelines.
- We have added this point to out limitations section, namely “However, as we only included guidelines published between 2017 and 2018, in English, and indexed in TRIP and Epistemonikos, our findings can neither be generalized to CPGs published in another time period, language or to guidelines indexed elsewhere.

However, in view of the effort involved, it is understandable that this route and this limitation were chosen. Also, a broader set of questions, including diagnosis and screening, would have been interesting, as there may be even greater deficiencies in evidence-based recommendations for these than for therapeutic questions.

- We have acknowledged this as a limitation in our discussion. Thanks for the comment.

Furthermore, the question arises why Cochrane Reviews per se seem to have a higher value than Non-Cochrane Reviews. There is no doubt that the methodological standard of Cochrane Reviews is generally very high, but there are also very good non-Cochrane Reviews (and also rather not so good Cochrane Reviews), so that it is quite understandable if these serve as a basis for a guideline recommendation, although a Cochrane Review exists.

- You are right that many non-Cochrane reviews are high quality, like the reviews by AHRQ or IQWIG as examples. In my opinion, it is authors that follow the methodological guidance by their affiliated organsations, and more experienced authors, that produce high quality evidence.

In addition to timeliness, contextual things may play a role here that are unlikely to be apparent without deeper analysis. In this context, a more in-depth discussion of the quality of the reviews would also have been desirable, but the authors themselves address this problem as a limitation.

- Yes thank you

Overall, this is a well-designed and well conducted analysis on a relevant topic. The conclusions can be derived from the analyzed data and limitations are addressed by the authors in the discussion.

- Thanks again for the discussion!
